# Perioperative neurocognitive disorders as a neuroimmune landscape disorder: microglial priming, state heterogeneity, and time-dependent neuroinflammation

**DOI:** 10.3389/fimmu.2026.1874245

**Published:** 2026-07-17

**Authors:** Mengjie Chen, Jiaqi Ning, Jiaxi Liu, Guanzheng Zhang, Siyi Xie, Ruyu Yan, Yuxuan Yang, Zhihao Ren, Xiang Li, Mingxue Zhou, Bin Li, Lingling Ding

**Affiliations:** 1 Department of Anesthesiology, Beijing Hospital of Traditional Chinese Medicine, Capital Medical University, Beijing, China; 2 Department of Acupuncture and Moxibustion, Beijing Pinggu District Hospital of Traditional Chinese Medicine, Beijing, China; 3 Beijing Hospital of Traditional Chinese Medicine, Capital Medical University, Beijing, China; 4 Beijing Institute of Chinese Medicine, Beijing, China; 5 Department of Acupuncture and Moxibustion, Beijing Hospital of Traditional Chinese Medicine, Capital Medical University, Beijing, China

**Keywords:** brain immune landscape, inflammatory resolution, microglial priming, neuroinflammation, perioperative neurocognitive disorders

## Abstract

Perioperative neurocognitive disorders (PND) are common and highly heterogeneous neurological complications in older and otherwise vulnerable surgical patients, with clinical manifestations ranging from delayed cognitive recovery to persistent postoperative cognitive decline. Although neuroinflammation is closely associated with the development of PND, an integrative framework is still lacking to explain how perioperative systemic immune activation leads to sustained central immune imbalance and cognitive dysfunction. Here, we propose that the brain immune landscape is a key determinant of susceptibility to PND. This landscape represents a baseline phenotype defined by central immune cell states, inflammatory activation thresholds, and resolution capacity. Within this microglia-centered framework, multiple perioperative factors may jointly drive maladaptive transitions in microglial states. We further discuss mitochondrial stress, glycolytic bias, epigenetic remodeling, non-coding RNA regulation, and trained immunity-like mechanisms, suggesting that these processes may serve as important drivers of sustained neuroimmune imbalance. This perspective supports time- and state-dependent, biomarker-guided intervention strategies aimed at preserving inflammatory resolution and enhancing perioperative cognitive resilience.

## 1 Introduction

Perioperative neurocognitive disorders (PND) comprise preexisting cognitive impairment, acute postoperative delirium, delayed neurocognitive recovery, and postoperative neurocognitive disorder (1). PND are increasingly recognized not as a transient consequence of anesthesia or surgery alone, but as a heterogeneous neurobiological response to perioperative stress, with trajectories ranging from acute fluctuation to persistent cognitive decline (2). Although neuroinflammation is strongly implicated (3), the extent of surgical injury or systemic inflammation alone cannot explain why comparable perioperative insults lead to markedly different cognitive outcomes.

Susceptibility to perioperative neuroinflammation may instead depend on the patient’s pre-existing glia-centered brain immune environment. Here, we define the brain immune landscape as a central immune ecosystem composed of microglia, astrocytes, brain microvascular endothelial cells, the blood-brain barrier/neurovascular unit (BBB/NVU), perivascular macrophages, infiltrating monocytes and T cells, and their intercellular communication networks (4). This landscape determines the threshold for inflammatory initiation, the mode of inflammatory amplification, and the capacity for resolution within the central nervous system.

Microglia occupy a central position in this landscape because they integrate peripheral inflammatory cues, barrier- and tissue-derived signals, neuronal activity, and synaptic information (5). However, postoperative microglial responses should not be reduced to a simple “resting versus activated” or M1/M2 dichotomy. Instead, microglia form a dynamic continuum of states shaped by aging, prior inflammatory exposure, metabolic stress, and comorbidities (6, 7). Primed microglia may show lower activation thresholds, exaggerated effector responses, altered metabolic programs, and impaired resolution capacity after perioperative challenge (8).

Recent advances in immunometabolism, epigenetic regulation, and innate immune memory suggest that perioperative neuroinflammation may involve durable microglial reprogramming rather than transient cytokine elevation alone (9). Mitochondrial dysfunction, glycolytic bias, lactate-related signaling, chromatin remodeling, non-coding RNA regulation, and trained immunity-like mechanisms may reset microglial response thresholds and constrain the return to homeostasis.

**Figure 1:**
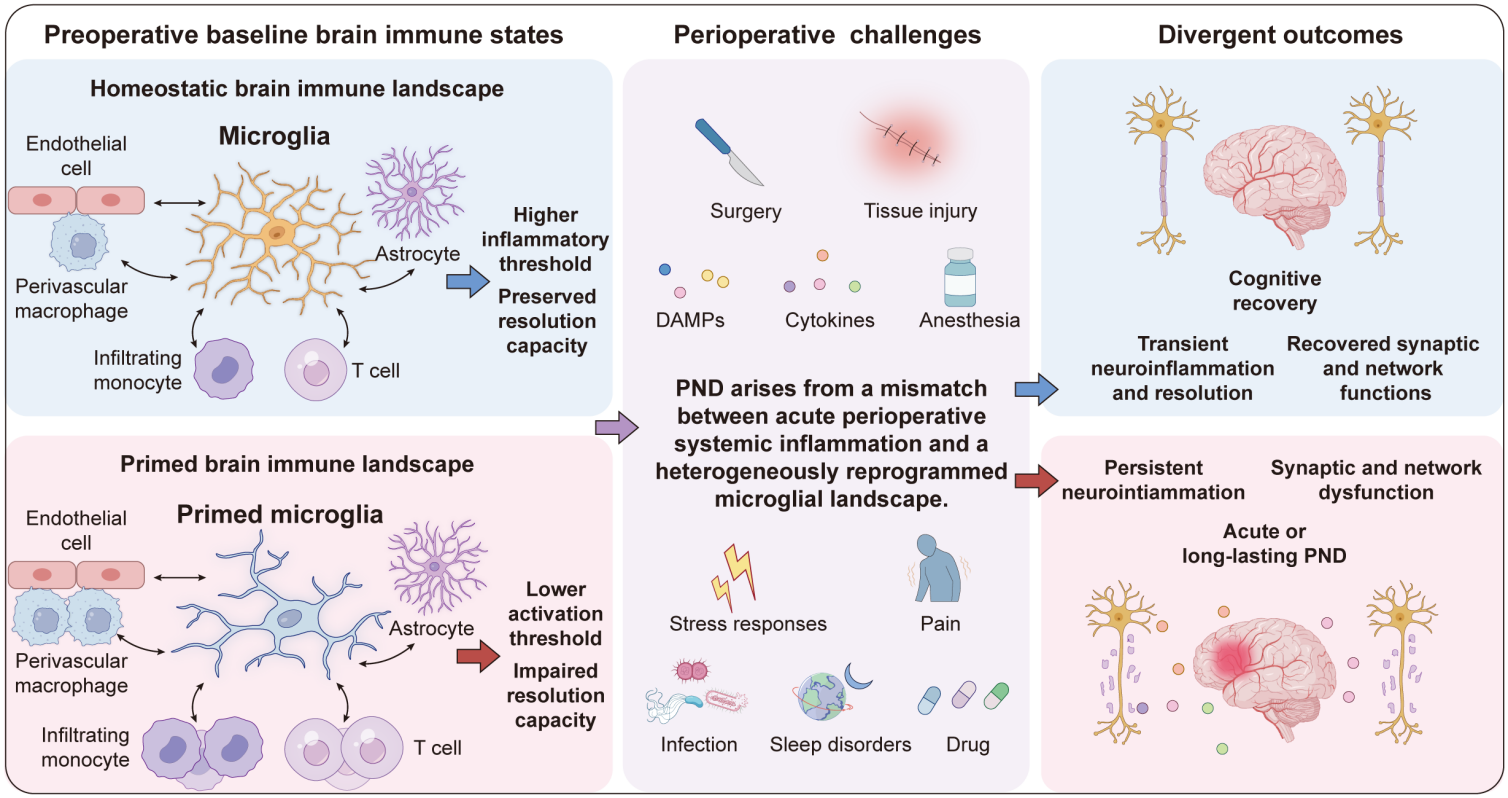
Conceptual framework linking brain immune landscape, microglial priming, and PND. This schematic shows how baseline brain immune status may influence perioperative neuroimmune responses and postoperative cognition. A homeostatic immune landscape, with balanced glial, vascular, and immune-cell interactions, supports a higher inflammatory threshold and effective resolution, favoring transient neuroinflammation, synaptic recovery, and cognitive recovery. In contrast, a primed immune landscape, marked by sensitized microglia, lower activation threshold, and impaired resolution, may respond maladaptively to perioperative challenges, leading to persistent neuroinflammation, synaptic/network dysfunction, and acute or long-lasting PND. DAMPs, damage-associated molecular patterns.

Building on this evidence, we propose that PND may arise from a mismatch between acute perioperative systemic inflammation and a primed, heterogeneously reprogrammed microglial landscape within the broader brain immune ecosystem. In this framework, microglia act as key integrators and amplifiers of immune signals between the periphery and the central nervous system, which may explain why some patients develop acute and long-lasting neurocognitive disorders after anesthesia and surgery (**Figure 1**). This review therefore synthesizes evidence on preoperative neuroimmune vulnerability, perioperative immune communication, microglial state transitions, metabolic and epigenetic reprogramming, and downstream synaptic and network dysfunction, aiming to inform time- and state-dependent perioperative interventions.

## 2 Preoperative neuroimmune vulnerability in the brain immune landscape

**Figure 2:**
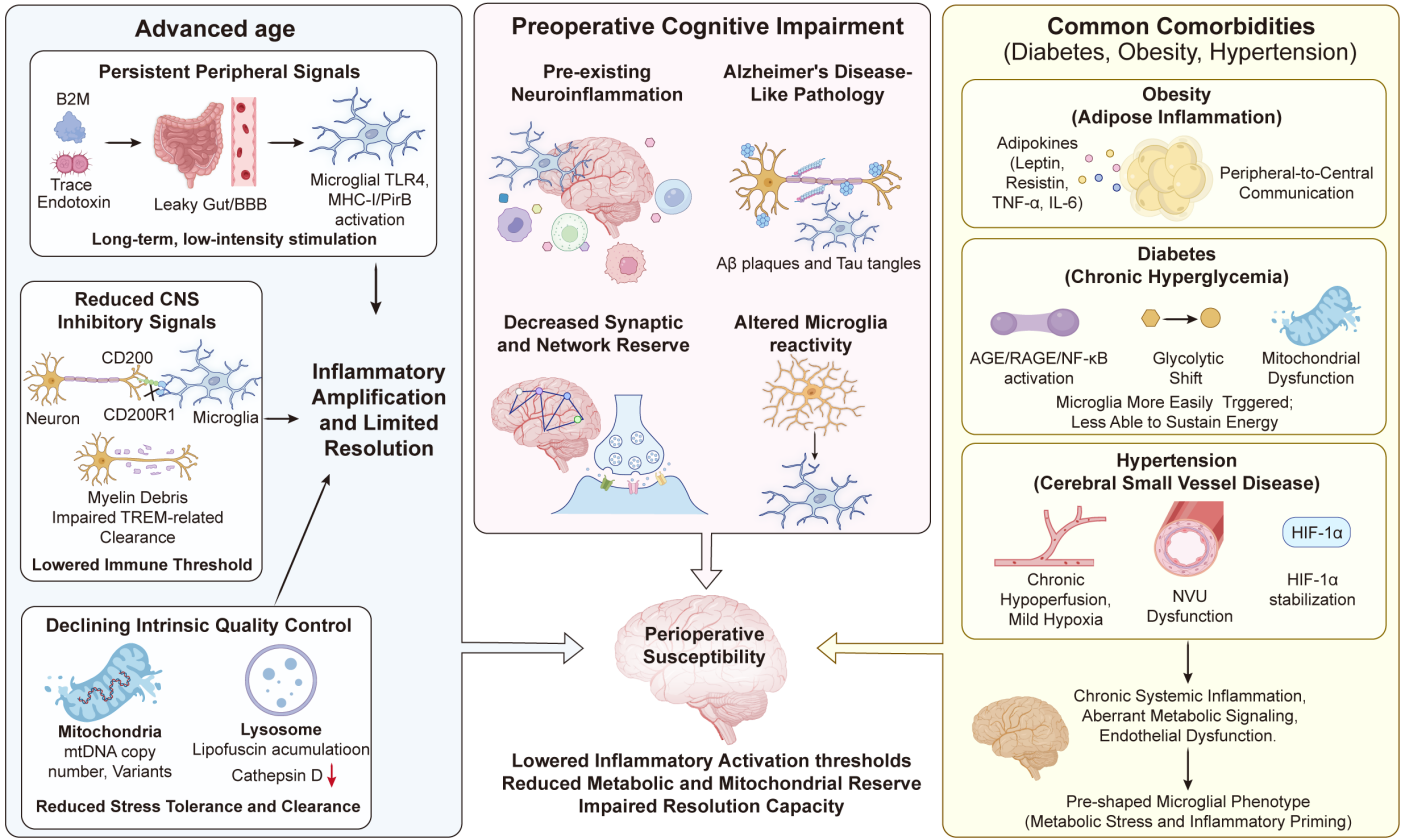
Advanced age, preoperative cognitive impairment, and comorbidities converge to increase perioperative susceptibility. This schematic shows how aging, preoperative cognitive impairment, and common comorbidities converge to reduce neuroimmune resilience before surgery. Aging-related inflammation, barrier leakage, impaired inhibitory and clearance pathways, cognitive-impairment-associated neuroinflammation and synaptic vulnerability, and comorbidity-driven metabolic and vascular stress collectively prime microglia, lower inflammatory thresholds, impair resolution capacity, and increase susceptibility to perioperative neurocognitive complications. Aβ, amyloid-β; AGE, advanced glycation end products; B2M, β-2 microglobulin; CD200R1, CD200 receptor 1; mtDNA, mitochondrial DNA; NVU, neurovascular unit; RAGE, receptor for advanced glycation end products; TREM, triggering receptor expressed on myeloid cells.

Susceptibility to perioperative neurocognitive disorders varies markedly across individuals and likely reflects differences in baseline neuroimmune vulnerability rather than a uniform response to surgical stress. Advanced age, preoperative cognitive impairment, and common systemic comorbidities are not merely epidemiological modifiers of risk (10). Instead, they reshape the preoperative brain immune landscape by altering microglial activation thresholds, effector programs, metabolic fitness, and resolution capacity, thereby influencing the magnitude and persistence of perioperative neuroinflammation (**Figure 2**).

### 2.1 Advanced age

Advanced age is the most consistently identified risk factor for PND. The aged brain is characterized by chronic low-grade inflammation, shortened microglial processes, elevated baseline pro-inflammatory cytokines, and increased expression of activation-associated markers such as MHC-II and CD68 (11). This vulnerability does not arise from a single pathway, but from the cumulative effects of persistent inflammatory input, weakened inhibitory control, and declining intracellular quality control.

Persistent low-intensity inflammatory signals can maintain microglia in a primed state before surgery. Aging-associated factors, such as β2-microglobulin (B2M) and gut-derived inflammatory cues, contribute to a chronic pro-inflammatory drive that favors baseline immune activation (12, 13). At the same time, reduced inhibitory signaling, exemplified by impairment of the CD200-CD200R1 axis, lowers the threshold for exaggerated microglial responses (14). Aging is also accompanied by impaired clearance of myelin debris, mitochondrial dysfunction, and lysosomal decline, which reduce stress tolerance and limit homeostatic recovery (15–19). Collectively, these changes create a preoperative state in which perioperative inflammatory challenge is more likely to trigger amplified and persistent neuroinflammation.

### 2.2 Preoperative cognitive impairment

Preoperative cognitive impairment is another important vulnerability factor for PND. Cohort studies and perioperative clinical investigations support that low baseline cognitive function, mild cognitive impairment (MCI), or dementia is associated with an increased risk of PND (20). Mechanistically, preoperative cognitive impairment may reflect pre-existing neuroinflammation, Alzheimer’s disease-like pathology, reduced synaptic and network reserve, and altered microglial reactivity.

Human translocator protein positron emission tomography (TSPO-PET) studies have shown that microglia-related neuroinflammatory signals are associated with Aβ deposition, tau burden, and poorer cognitive performance (21). In parallel, studies of microglial immunometabolism further suggest that cognitive impairment-related pathology may be accompanied by glycolytic bias, abnormal lipid handling, limited Aβ clearance, and impaired phagocytic and reparative functions (22, 23). Collectively, these changes may indicate a preoperative brain immune landscape in which microglia have a lower inflammatory activation threshold, reduced metabolic reserve, and limited capacity for inflammatory resolution, thereby increasing susceptibility to PND.

### 2.3 Common comorbidities: diabetes, obesity, and hypertension

Common perioperative comorbidities, including diabetes, obesity, and hypertension, should not be viewed merely as statistical correlates, but rather as mechanistic drivers of neuroimmune vulnerability. Obesity promotes chronic peripheral inflammation and sustains adipokine signaling, including leptin and resistin, thereby influencing brain immune states through peripheral-to-central communication pathways (24). Diabetes, by contrast, imposes inflammatory-metabolic stress through chronic hyperglycemia and AGE/RAGE/NF-κB-related signaling, which may increase the risk of delayed inflammatory resolution (25–27). Hypertension-associated cerebral small vessel disease further weakens the fine regulation of the neurovascular unit, placing brain tissue in a state of chronic hypoperfusion and mild hypoxia (28). Under hypoxic conditions, hypoxia-inducible factor 1α (HIF-1α) becomes stabilized, thereby promoting a glycolytic shift in microglia and enhancing the transcriptional activity of pro-inflammatory genes such as IL-1β (29).

Taken together, advanced age, preoperative cognitive impairment, and common systemic comorbidities should not be regarded as isolated modifiers of perioperative risk. Rather, they converge on a vulnerable brain immune landscape characterized by lowered inflammatory activation thresholds, reduced metabolic and mitochondrial reserve, impaired resolution capacity, and increased BBB/NVU vulnerability. In this preoperative state, perioperative immune challenge is more likely to elicit exaggerated and persistent neuroinflammatory responses, thereby increasing susceptibility to PND.

## 3 Perioperative inputs and communication pathways that disturb the brain immune landscape

**Figure 3:**
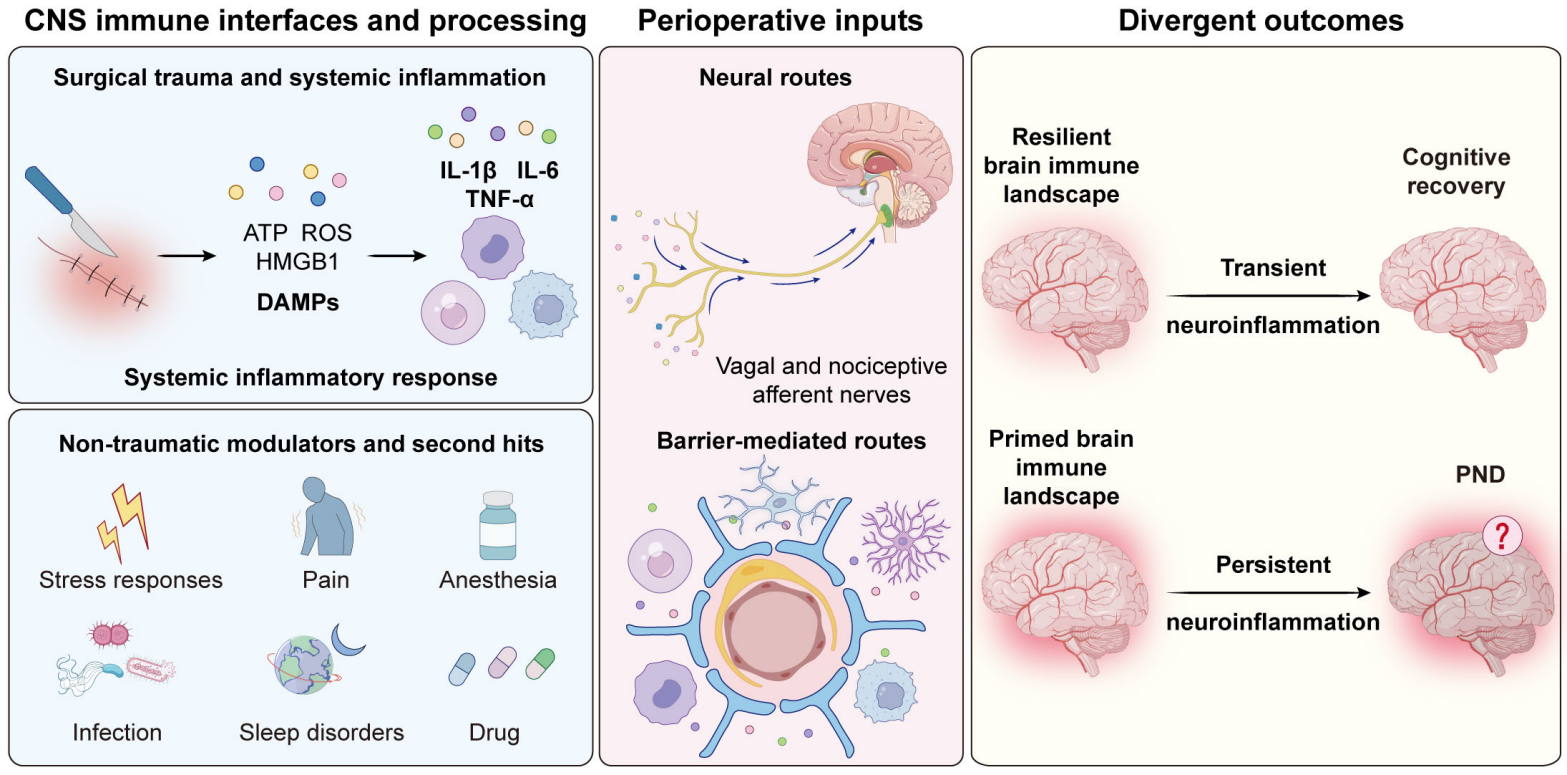
CNS immune interfaces translate perioperative insults into divergent neurocognitive outcomes. This schematic shows how perioperative inflammatory and non-inflammatory inputs are processed through CNS immune interfaces to shape postoperative outcomes. Surgical trauma triggers systemic inflammation, with release of ATP, ROS, HMGB1, DAMPs, and cytokines including IL-1β, IL-6, and TNF-α. Stress, pain, anesthesia, infection, sleep disruption, and drug exposure may act as second hits. These signals reach the brain via neural routes, such as vagal and nociceptive afferents, and barrier-mediated immune–vascular routes. In a resilient brain immune landscape, they induce transient neuroinflammation and cognitive recovery, whereas in a primed landscape they may drive persistent neuroinflammation and increase PND risk. ATP, adenosine triphosphate; DAMPs, damage-associated molecular patterns; HMGB1, high-mobility group box 1; ROS, reactive oxygen species.

The perioperative period is a defined time window. During this period, systemic inflammation, neural stress, drug exposure, and behavioral disruption act together on the central nervous system (CNS). Surgical trauma is the main initiating event. Multiple perioperative inputs are transmitted through regulated peripheral-to-brain communication pathways. They are then interpreted within the pre-existing brain immune landscape. This interaction helps determine whether CNS immune activation resolves smoothly or shifts toward persistent neuroinflammation (**Figure 3**).

### 3.1 Surgical trauma and systemic inflammatory inputs

Surgical trauma is a major trigger of sterile systemic inflammation. Tissue injury, organ manipulation, and ischemia-reperfusion release damage-associated molecular patterns (DAMPs) such as extracellular ATP, mitochondrial DNA, high mobility group box 1 (HMGB1), and heat shock proteins, thereby activating pattern-recognition receptors and downstream NF-κB, inflammasome, cytokine, complement, and leukocyte-recruitment pathways (30–32). This systemic response is accompanied by increased IL-1β, IL-6, TNF-α, CCL2, C-reactive protein, and hypothalamic-pituitary-adrenal and sympathetic activation (33). However, inflammatory burden alone does not determine cognitive outcome: comparable surgical stress can be followed by normal recovery, transient delirium, or persistent cognitive decline (34–36). Thus, systemic inflammation is a key upstream trigger of PND, but disease heterogeneity depends on how this input is interpreted by the pre-existing brain immune landscape.

### 3.2 Nontraumatic perioperative modulators: anesthesia, drug exposure, pain and stress responses, and postoperative second hits

In addition to tissue injury, several nontraumatic perioperative factors can alter neuroimmune susceptibility. Anesthesia should not be viewed as an isolated cause of PND. Rather, it should be understood as a context-dependent modulator of brain immune responses. General anesthetics can affect neuronal activity, neurotransmitter release, brain metabolism, mitochondrial function, and network oscillations. They may also change the threshold for CNS immune responses by influencing neuron-microglia communication, synaptic activity, and region-specific circuit states (37–39). In a healthy or resilient brain, these changes are often reversible. In brains affected by aging, cognitive impairment, metabolic abnormalities, or vascular comorbidities, anesthetic exposure may interact with surgical inflammation and promote a shift of microglia from homeostasis toward a hyperresponsive state (40, 41).

Perioperative drug exposure may also influence neuroimmune stability. Sedatives, opioids, anticholinergic drugs, corticosteroids, antibiotics, and vasoactive agents can affect arousal, neurotransmission, sleep architecture, gut-immune signaling, vascular regulation, and metabolic homeostasis (42–45). Their effects are not uniformly harmful or protective. They depend on dose, timing, drug combinations, patient susceptibility, and interactions with surgical stress. For example, inadequate analgesia can sustain nociception and sympathetic activation, thereby prolonging inflammatory input. In contrast, excessive sedation, anticholinergic burden, or unstable hemodynamic management may disrupt arousal networks, BBB/NVU homeostasis, and cerebral metabolic balance, increasing the risk of delirium or cognitive fluctuation (46, 47).

Pain, stress, and behavioral disruption represent persistent postoperative inputs. Sustained nociceptive signaling can activate spinal and supraspinal immune pathways and enhance sympathetic and hypothalamic-pituitary-adrenal axis responses (48, 49). Sleep fragmentation, circadian disruption, immobilization, sensory deprivation, and unfamiliar environments may further weaken inflammatory resolution and neural network stability (50–52). In patients with pre-existing microglial priming or BBB/NVU vulnerability, these factors may not independently trigger PND, but they can act as persistent amplifiers that delay the return to homeostasis.

Postoperative second hits may further determine whether perioperative neuroimmune responses resolve or become persistent. Infection, hypoxia or hypoperfusion, anemia, hyperglycemia, renal dysfunction, electrolyte imbalance, transfusion-related inflammation, and repeated procedures can re-amplify systemic inflammation or metabolic stress (53). In a primed or vulnerable brain immune landscape, even mild secondary injury may lower the threshold for microglial responses, delay inflammatory resolution, and promote sustained neuroimmune activation (54). Thus, PND should not be attributed to surgical trauma alone. It is better understood as the result of surgical inflammation, anesthesia and drug exposure, pain and stress responses, behavioral disruption, and postoperative second hits acting together on the pre-existing brain immune landscape.

### 3.3 Convergence of perioperative inputs on CNS immune interfaces

Perioperative inflammatory signals can affect CNS function through neural, barrier-mediated, and cellular routes without necessarily entering the brain parenchyma (55). Vagal and nociceptive afferents rapidly convey visceral, inflammatory, and pain-related signals to brainstem autonomic and neuroendocrine circuits (56, 57). At CNS barrier interfaces, including the BBB/NVU, circumventricular organs, and the choroid plexus-cerebrospinal fluid axis, cytokines and DAMPs activate endothelial and barrier-associated receptors, including IL-1R1, TNFR1, and gp130-associated receptors, inducing COX-2/PGE2 signaling, adhesion molecule expression, chemokine release, and barrier changes (58–60). Increased ICAM-1, VCAM-1, and CCL2 can recruit CCR2-positive monocytes, while Th17/Treg imbalance and CD8-positive T-cell-derived IFN-γ further amplify neuroimmune responses (61–64). These interfaces therefore act as immune-sensing platforms: in resilient brains the response is transient, whereas microglial priming, BBB/NVU dysfunction, impaired mitochondrial reserve, or limited resolution capacity can convert similar inputs into persistent neuroinflammation.

## 4 Neuroimmune state transitions after perioperative disturbance: a microglia-centered multicellular model

**Figure 4:**
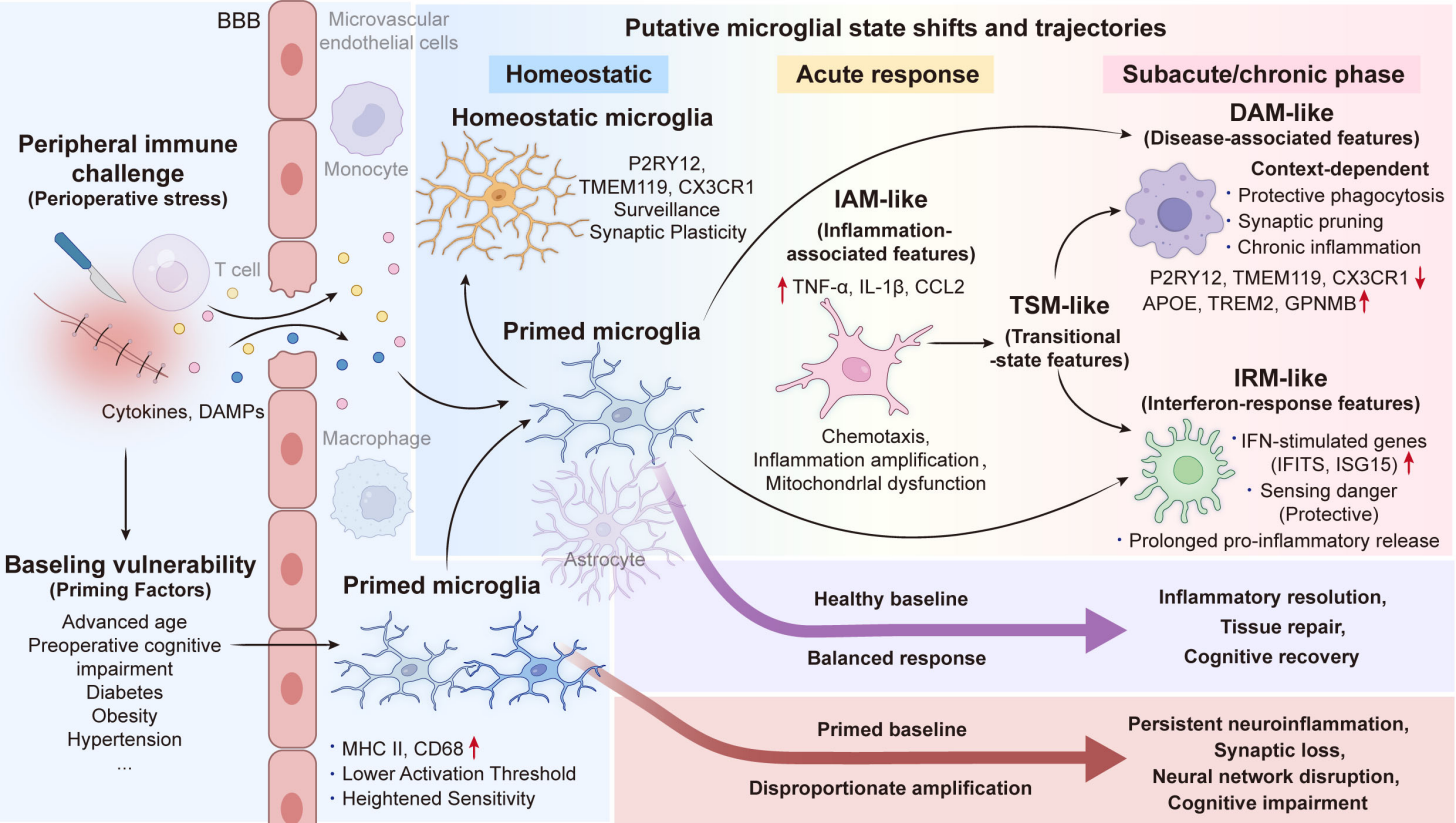
Putative microglial state shifts may be associated with perioperative immune challenge and divergent cognitive outcomes. This schematic presents a proposed framework in which perioperative immune challenges may interact with baseline vulnerability to influence microglial state distributions across the blood-brain barrier. Surgical stress and systemic inflammation may promote cytokine and DAMP release, whereas aging, preoperative cognitive impairment, diabetes, obesity, hypertension, and other priming factors may increase susceptibility. In a homeostatic landscape, microglia tend to preserve surveillance and synaptic-support programs, with maintained P2RY12, TMEM119, and CX3CR1 expression. After perioperative stress, microglia may exhibit inflammatory or transitional features resembling IAM-like, TSM-like, DAM-like, or IRM-like states, although their timing and functional roles in PND remain incompletely defined. A resilient baseline may favor inflammatory resolution and cognitive recovery, whereas a primed baseline may be associated with persistent neuroinflammation, synaptic/network disruption, and cognitive impairment. APOE, apolipoprotein E; CX3CR1, C-X3-C motif chemokine receptor 1; DAM-like, disease-associated microglia-like state; DAMPs, damage-associated molecular patterns; IAM-like, inflammation-associated microglia-like state; IFN, interferon; IRM-like, interferon-response microglia-like state; P2RY12, purinergic receptor P2Y12; PND, perioperative neurocognitive disorder; TMEM119, transmembrane protein 119; TREM2, triggering receptor expressed on myeloid cells 2; TSM-like, transitional-state microglia-like state.

Perioperative signals transmitted through neural, vascular, and cellular interfaces ultimately reshape the immune state of the CNS. In this process, microglia hold a central position. They can integrate peripheral inflammatory signals, barrier-derived signals, neuronal activity signals, and tissue-derived danger signals (65). However, perioperative neuroinflammation should not be understood as a microglia-autonomous process. Astrocytes, endothelial cells, BBB/NVU components, perivascular macrophages, infiltrating monocytes, and T cells also shape the direction, duration, and functional consequences of microglial responses (66). Therefore, PND-related neuroinflammation should be understood as a dynamic multicellular state transition (**Figure 4**). It should not be viewed as a simple increase in inflammatory markers or a binary M1/M2 polarization (67, 68).

### 4.1 Initial conditions: homeostatic and primed microglia before perioperative exposure

The microglial response to perioperative stress largely depends on the preoperative microglial state. In a resilient brain immune landscape, homeostatic microglia maintain immune surveillance, synaptic regulation, debris clearance, and local inhibitory control (69). Homeostatic microglia usually express core homeostasis-related markers, such as P2RY12, TMEM119, and CX3CR1. They also maintain immune surveillance, synaptic regulation, and anti-inflammatory control through neuron-microglia communication axes, including CD200-CD200R and CX3CL1-CX3CR1 (14, 70). Under these conditions, perioperative immune stimulation may induce a transient inflammatory response. This response may then enter resolution and return to homeostasis.

Microglia may already enter a primed state before surgery. Primed microglia are not fully activated in the traditional sense. Rather, priming represents an initial condition with a low activation threshold and high responsiveness. It is characterized by increased sensitivity to DAMPs, cytokines, neuroendocrine stress signals, anesthetic exposure, and postoperative second hits (71, 72). This concept was initially derived mainly from models of aging, prion disease, traumatic brain injury, LPS stimulation, and other forms of neuroinflammation (73–76). In recent years, perioperative studies have provided more direct support for this concept. For example, in aged mice, age-related loss of microglial Mef2C can induce a primed phenotype, amplify postoperative neuroinflammatory responses, and promote the development of PND (77).

Therefore, homeostatic and primed microglia should be viewed as distinct initial conditions for perioperative neuroimmune responses. They determine how perioperative inputs are interpreted, how strong the microglial response becomes, and whether the subsequent response remains adaptive or shifts toward maladaptation.

### 4.2 Acute postoperative state transitions: loss of homeostatic programs and expansion of IAM/TSM-like states

Microglial responses during the acute postoperative phase should not be simply described as a shift from “resting” to “activated” states. They are more likely to involve rapid remodeling of cellular state composition and intercellular communication. Current single-cell evidence in PND remains limited. However, a recent study using an aged surgical model provided important insight. Single-cell transcriptomic sequencing of the hippocampus at 24 h after surgery showed changes in microglial subgroup composition. In this study, IAM-like and TSM-like microglial populations expanded. This was accompanied by remodeling of glial-vascular communication related to signaling axes such as TNF, CSF1, and ICAM1 (40).

In addition to the expansion of reactive states, postoperative microglia may also show weakened homeostasis-like molecular features. Studies have shown that perioperative stress can downregulate the expression of microglial homeostasis-related markers, including P2RY12, TMEM119, and CX3CR1 (40, 65). This suggests that homeostasis-related transcriptional features may be impaired. Functionally, these changes may affect microglial immune surveillance, synaptic maintenance, clearance of apoptotic cells, and inflammatory resolution. Therefore, PND should not be explained only as excessive inflammation. It may also involve insufficient capacity of microglia to maintain homeostasis.

At the same time, expansion of IAM-like states may promote early postoperative inflammatory amplification. During the early postoperative period, IAM-like cells may show increased signals related to inflammatory factors and chemokines, such as TNF, IL-1β, and CCL2. They may amplify local inflammatory responses through TNF-dominated glial communication, non-coding RNA (ncRNA), CCL2/CCR2-related recruitment of peripheral immune cells, and BBB/NVU injury (40, 78). TSM-like populations should be understood more as an intermediate state. They suggest that microglia are shifting from homeostasis toward inflammation-related, disease-associated, or repair-related programs, rather than representing a fixed terminal phenotype (79).

Therefore, based on the currently limited single-cell and transcriptomic evidence, acute postoperative microglial changes can be provisionally summarized within a “state imbalance” framework. In some models, IAM-like and TSM-like states expand, while homeostasis-related molecular features are weakened. This framework better captures the heterogeneity of microglial responses than the traditional M1/M2 model. However, it remains dependent on the model, time window, and baseline state. Notably, not all perioperative transcriptomic studies support a model of uniform pro-inflammatory activation of microglia after surgery. For example, microglia in aged mice may transiently show a homeostasis-like or hyporesponsive molecular phenotype after surgery. This finding suggests that postoperative microglial responses are strongly context-dependent (80).

### 4.3 Subacute and persistent trajectories: DAM-like and IRM-like states, repair, resolution, or maladaptive persistence

After the acute postoperative phase, microglial state trajectories may diverge. In some cases, reactive microglia may shift toward debris clearance, tissue repair, and inflammatory resolution. However, when the baseline immune landscape is already primed or vulnerable, reactive states may persist and shift toward a maladaptive inflammatory trajectory. This divergence helps explain why some patients recover rapidly, whereas others develop persistent or fluctuating cognitive decline.

DAM-like programs may be involved in the subacute postoperative phase (40). DAM-like cells are usually characterized by downregulation of homeostatic markers, such as P2RY12, TMEM119, and CX3CR1, together with upregulation of disease-associated genes, such as APOE, TREM2, and GPNMB (81). Their functions are context-dependent. On the one hand, DAM-like responses may help clear debris, remove damaged structures, and support tissue adaptation (82). On the other hand, if DAM-like activity is excessive or persistent, it may promote abnormal phagocytosis, synaptic loss, chronic low-grade inflammation, and network instability (83, 84). Therefore, DAM-like states should not be simply classified as protective or harmful. Their effects depend on the time window, stimulus intensity, brain region, and capacity for inflammatory resolution.

Interferon-response microglia-like states (IRM-like) may contribute to persistent neuroimmune changes in PND (18, 85). IRM-like cells are characterized by increased expression of interferon-stimulated genes, including IFIT family members and ISG15 (86). Postoperative IFN-β and IFN-γ are associated with sustained microglial activation (85). Interferon-related responses may enhance danger-signal sensing and host defense-like programs. However, if interferon signaling persists, it may increase IL-1β, CXCL10, immune cell recruitment, and inflammatory tissue injury (87). In a vulnerable brain immune landscape, persistent IRM-like activity may allow CNS inflammation to continue even after peripheral inflammatory markers have recovered.

At present, DAM-like and IRM-like programs have been characterized in several neuropathological contexts. However, their timing and functions under perioperative stress remain unclear. The key issue in PND-related neuroinflammation is not whether a specific microglial state appears. Rather, it is whether state transitions are transient, reversible, and able to restore homeostasis. If IAM-like, DAM-like, or IRM-like programs persist, they may impair the recovery of homeostasis, promote abnormal synaptic remodeling, and enhance glial-vascular or peripheral immune interactions. These changes may further drive inflammatory amplification and cognitive injury (40).

### 4.4 Multicellular interactions that shape microglial trajectories

Although microglia are key integrators of perioperative neuroimmune signals, their state transitions are not determined by microglia alone. Astrocytes, endothelial cells, BBB/NVU components, perivascular macrophages, and infiltrating monocytes and T cells jointly shape postoperative microglial trajectories. Therefore, the perioperative brain immune landscape should be understood as a multicellular regulatory network rather than a microglia-autonomous process. This view is also consistent with studies of delirium and acute cognitive impairment. In these conditions, microglia, astrocytes, oligodendrocytes, BBB dysfunction, and white matter alterations jointly contribute to acute brain dysfunction and cognitive fluctuations (88).

Within this network, astrocytes can influence postoperative inflammatory responses by regulating cytokines, glutamate homeostasis, metabolic support, complement signaling, and BBB integrity (89). The single-cell atlas of the aged hippocampus also suggests that postoperative cognitive impairment is accompanied by disruption of the neuron-glia system among neurons, microglia, astrocytes, and oligodendrocytes. Pathways such as PGD2/DP and complement signaling may be involved in the disruption of synaptic and myelin homeostasis (67). Peripheral immune cells may also further sustain inflammatory or interferon-related microglial programs through Th17/Treg imbalance or IFN-γ derived from CD8-positive T cells (63, 64).

Therefore, IAM-, DAM-, and IRM-like states should not be viewed as isolated microglial phenotypes. Instead, they are dynamic states formed through continuous interactions among resident glial cells, vascular interfaces, and infiltrating immune cells. This multicellular framework helps explain why perioperative neuroinflammation may persist even after systemic inflammatory markers gradually recover.

## 5 Mechanisms of perioperative microglial reprogramming in the brain

**Figure 5:**
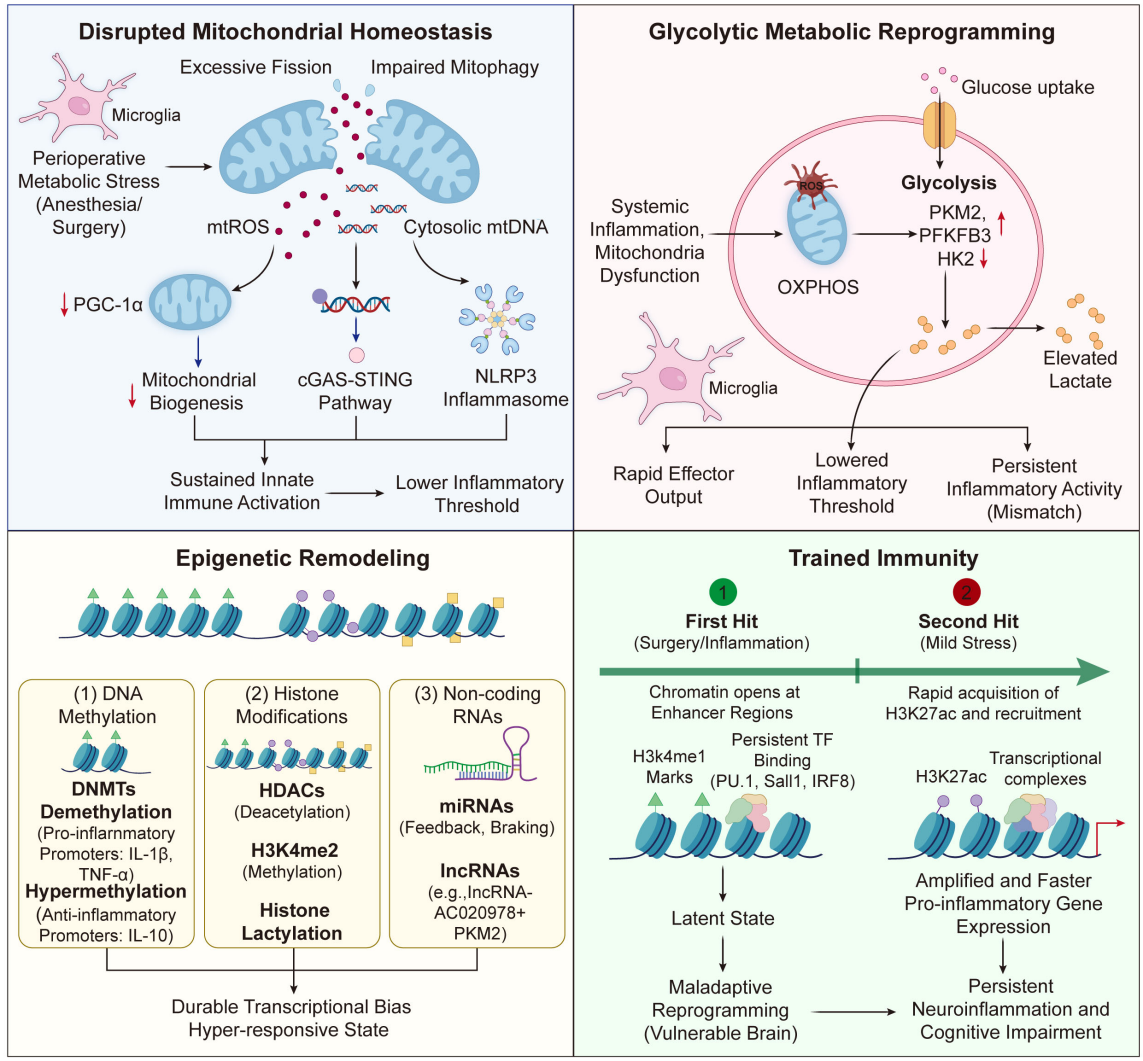
Mitochondrial, metabolic, and epigenetic mechanisms, together with trained immunity, may sustain microglial hyper-responsiveness after perioperative stress. This figure illustrates four interconnected mechanisms by which perioperative metabolic stress and systemic inflammation reprogram microglia and sustain neuroinflammation: mitochondrial dysfunction releases mtROS and mtDNA to activate cGAS-STING and NLRP3 and lower the activation threshold; a shift from oxidative phosphorylation to glycolysis increases lactate and prolongs inflammatory output; epigenetic remodeling biases microglia toward pro-inflammatory transcription; and trained immunity may create a memory-like state in which later mild stress triggers an exaggerated response, contributing to neuroinflammation and cognitive impairment. DNMTs, DNA methyltransferases; HDACs, histone deacetylases; H3K4me1, histone H3 lysine 4 monomethylation; H3K4me2, histone H3 lysine 4 dimethylation; H3K27ac, histone H3 lysine 27 acetylation; lncRNAs, long non-coding RNAs; miRNAs, microRNAs; mtDNA, mitochondrial DNA; mtROS, mitochondrial reactive oxygen species.

Microglial priming and state heterogeneity in perioperative neuroinflammation are unlikely to be transient phenomena. They may be maintained by durable processes of cellular reprogramming that reset response thresholds and constrain inflammatory resolution. Current evidence suggests that disrupted mitochondrial homeostasis, glycolytic metabolic reprogramming, epigenetic remodeling, and trained immunity jointly shape microglial effector outputs and inflammatory memory (**Figure 5**) (90). These processes provide a biological basis for why an acute systemic insult can, under specific backgrounds, precipitate long-lasting and maladaptive neuroimmune responses.

### 5.1 Disrupted mitochondrial homeostasis

Perioperative metabolic stress can impair microglial mitochondrial homeostasis, increase ROS production, and amplify innate inflammatory signaling (39, 91, 92). Anesthesia and surgery may promote excessive mitochondrial fission, increasing the likelihood of cytosolic mtDNA leakage. Cytosolic mtDNA and mtROS can then function as danger signals that activate cGAS-STING and NLRP3-related pathways, thereby lowering the threshold for inflammatory activation (93, 94). In parallel, reduced PGC-1α activity may impair mitochondrial biogenesis and respiratory chain gene expression, contributing to ATP insufficiency and limited recovery capacity (95–97).

Perioperative studies also suggest that the direction of mitochondrial quality-control responses may depend on the timing and severity of injury. In exploratory laparotomy models, surgical stress increases mitophagy and is accompanied by elevated cGAS-STING signaling, whereas PINK1 knockdown disrupts mitophagy, promotes cytosolic mtDNA accumulation, and strengthens innate immune activation (98, 99). These findings indicate that early mitophagy may initially limit damage-signal propagation. However, when lysosomal function or energy supply is insufficient, mitophagy may shift from compensatory activation to impaired flux. Persistent retention of dysfunctional mitochondria then sustains mtDNA, mtROS, cGAS–STING, and NLRP3 signaling, creating microglial subsets with lower inflammatory thresholds and delayed resolution after secondary stress.

### 5.2 Glycolytic metabolic reprogramming

Mitochondrial dysfunction and systemic inflammation can push perioperative microglia from oxidative phosphorylation toward glycolysis. This glycolytic bias supports rapid inflammatory effector output but may also reduce metabolic efficiency, lower response thresholds, and prolong inflammatory activity (100, 101). Clinical and experimental findings support this model. In older patients with hip fracture, targeted CSF metabolomics showed that lactate levels were associated with postoperative delirium (102). In older trauma patients undergoing general anesthesia, early postoperative serum lactate was an independent predictor of PND (103). In animal models, surgical trauma can induce pro-inflammatory microglial responses and upregulate PKM2, linking glycolytic activation to cognitive impairment (104–106). A perioperative pattern characterized by PFKFB3 upregulation and HK2 downregulation may reflect a metabolic mismatch. On the one hand, microglia are driven toward glycolysis to sustain inflammatory output. On the other hand, limited substrate entry or mitochondrial reserve may impose compensatory stress. This mismatch may lock microglia into a state of persistent inflammatory activity with reduced resolution capacity. Thus, glycolytic reprogramming is not merely a marker of activation; it may actively maintain low-threshold, long-output microglial states that favor persistent neuroinflammation (107).

### 5.3 Epigenetic remodeling

Compared with acute metabolic changes, epigenetic remodeling can impose more durable transcriptional bias. DNA methylation, histone modifications, and non-coding RNAs may reshape chromatin accessibility and transcriptional persistence, thereby lowering microglial response thresholds and delaying resolution (9, 108, 109).

Perioperative studies indicate that surgical stress can alter methylation at inflammation-related genes (110, 111). In microglia, reduced promoter methylation of IL-1β, IL-6, and TNF-α, together with increased methylation at the IL-10 promoter, suggests an asymmetric epigenetic pattern: pro-inflammatory genes become easier to activate, whereas anti-inflammatory braking becomes harder to engage (112, 113). This pattern resembles a priming-like state and may increase the amplitude of responses to postoperative second hits. Histone modifications further contribute to inflammatory persistence. In PND models, anesthesia or surgery has been associated with increased hippocampal HDAC1, HDAC2, and HDAC8 enrichment and enhanced microglial inflammatory responses (114–117). Class I HDAC inhibitors can suppress inflammatory pathways and partially reverse activated phenotypes, suggesting that a high-HDAC state may support inflammatory amplification (116, 118). Other chromatin marks, such as H3K4me2 at inflammatory loci, may facilitate NLRP3/caspase-1/IL-1β signaling (119). Histone lactylation may also link glycolytic metabolism to epigenetic regulation through the YTHDF3/PRDX3 axis and NLRP3-related microglial pyroptosis (120). These findings suggest that lactate-related pathways may contribute not only to metabolic stress but also to the persistence and reactivation of inflammatory programs.

Non-coding RNAs provide another layer of threshold control. Several microRNAs can dampen neuroinflammatory output and protect against surgical trauma-induced cognitive decline (121–123), indicating that microglia retain endogenous braking circuits. Conversely, lncRNA-AC020978 can interact with PKM2 to promote glycolysis and enhance inflammatory responses (104). Because ncRNA effects are strongly dependent on timing, stimulus intensity, and model context, they should be interpreted as modulators of microglial state transitions rather than fixed markers of injury (124). Overall, epigenetic and ncRNA regulation may help explain why perioperative microglia remain easily reactivated even after the initial inflammatory trigger has subsided.

### 5.4 Trained immunity

Trained immunity provides a plausible framework for understanding how a brief perioperative insult may produce long-lasting neuroimmune consequences (125). Microglia can acquire memory-like response features after prior inflammatory exposure, mainly through persistent changes in chromatin accessibility and epigenetic marks rather than antigen-specific receptors (126). In the perioperative context, this concept remains partly inferential, but it is useful for explaining why primed or vulnerable brains may respond disproportionately to mild postoperative second hits.

After an initial exposure to surgical stress or peripheral inflammation, regulatory regions of inflammatory genes such as TNF and IL-6 may acquire enhancer-associated marks. H3K4me1 can maintain chromatin in a poised configuration and favor the binding of transcription factors such as PU.1, Sall1, and IRF8 (127). During a later mild infection, hypoxia, metabolic disturbance, or stress exposure, these latent enhancers may rapidly gain H3K27ac and recruit transcriptional complexes, increasing the speed and amplitude of pro-inflammatory gene expression (128, 129).

This form of microglial memory is not necessarily pathological. Under physiological conditions, it may enhance host defense (130). In aged, metabolically stressed, or otherwise vulnerable brains, however, the same mechanism may shift toward maladaptive reprogramming. The result is a lower activation threshold, stronger inflammatory amplification, and constrained homeostatic recovery. By linking metabolic stress, epigenetic persistence, and secondary-hit hypersensitivity, trained immunity provides a mechanistic explanation for delayed resolution and persistent neuroinflammation after surgery.

## 6 Neuroinflammation meets synaptic and network dysfunction

**Figure 6:**
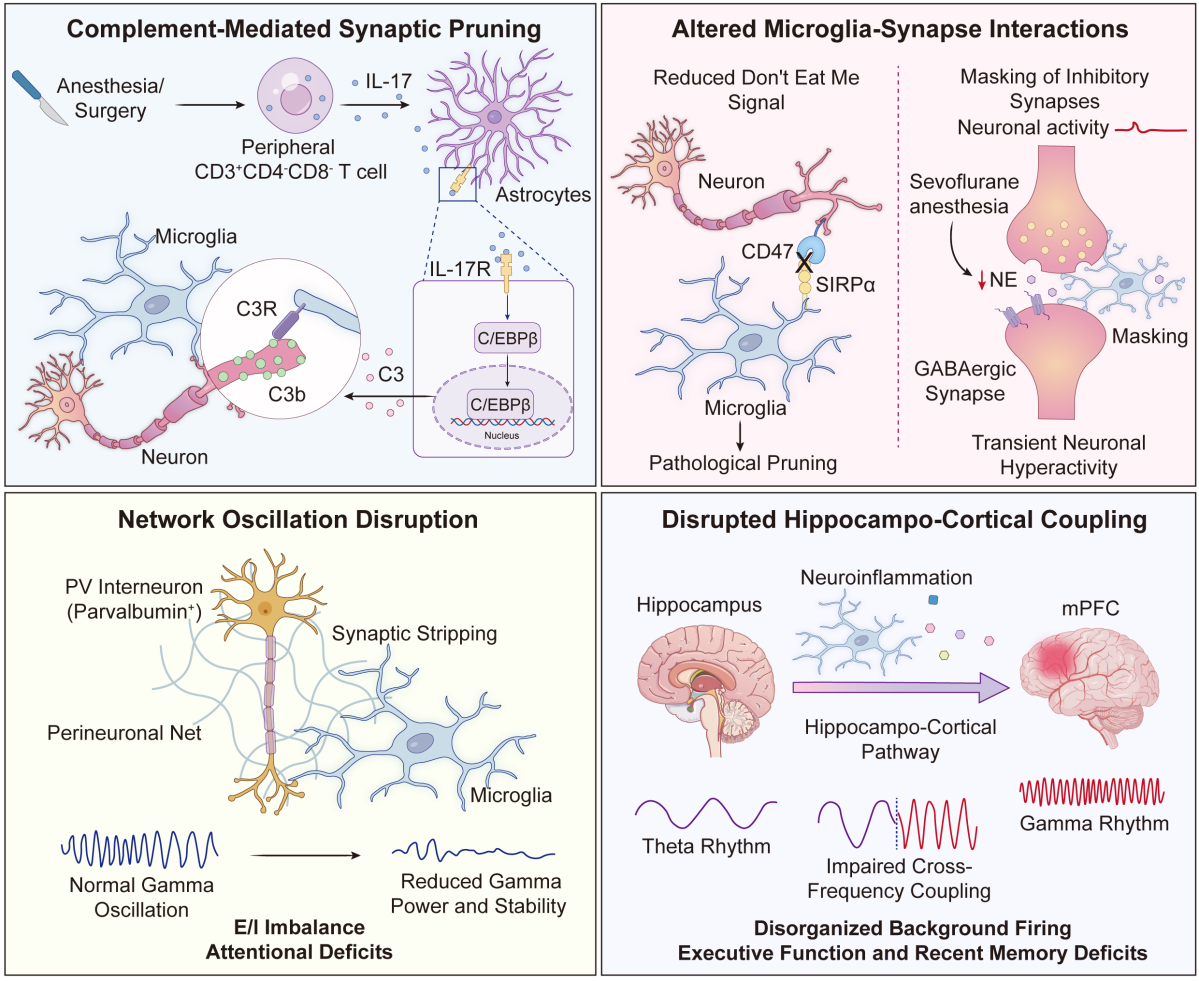
Microglia-synapse interactions and circuit disruption after perioperative immune activation. This schematic summarizes proposed pathways by which perioperative immune activation may affect synaptic integrity and neural circuit function. Anesthesia and surgery may promote IL-17-related T cell responses, astrocytic IL-17R signaling, and complement activation involving C3/C3b and C3aR, potentially enhancing microglia-mediated synaptic pruning. Altered CD47-SIRPα “don’t eat me” signaling and sevoflurane-associated reductions in norepinephrine may further disturb microglia-synapse interactions. These changes may contribute to PV interneuron dysfunction, reduced gamma oscillation stability, excitation/inhibition imbalance, impaired hippocampo-cortical coupling, and deficits in attention, executive function, and recent memory. C3, complement component 3; C3b, complement component 3b; C3aR, complement C3a receptor; E/I, excitation/inhibition; NE, norepinephrine; PV, parvalbumin.

Microglial priming and reprogramming set the inflammatory threshold of the perioperative brain, but cognitive impairment becomes clinically evident when immune imbalance disrupts synaptic processing and network coordination. In this process, microglia link inflammatory intensity, persistence, and resolution failure to synaptic remodeling and circuit instability. Perioperative evidence is strongest for complement-mediated synaptic loss and CD47-SIRPα-related engulfment control, whereas network oscillation mechanisms should be interpreted as plausible extensions supported by perioperative and broader neuroinflammation models (**Figure 6**) (131).

### 6.1 Complement-mediated synaptic remodeling

Complement-dependent synaptic tagging provides a direct route by which perioperative inflammation may be converted into synaptic loss. Prolonged sevoflurane anesthesia increases hippocampal C1qa and C3 expression in aged rats, promotes C3b deposition, and enhances microglial engulfment of synapses, leading to synaptic loss and functional impairment (132). In fracture-surgery models, astrocyte-derived C3 preferentially tags presynaptic excitatory structures and activates microglial C3aR-related signaling, increasing phagocytic activity and contributing to memory impairment (133).

Peripheral immune inputs may further amplify this pathway. Exploratory laparotomy can induce hippocampal migration of CD3-positive CD4-negative CD8-negative double-negative T cells. Their IL-17 activates IL-17R-CEBPβ signaling in astrocytes and promotes C3 production (134). These findings support a perioperative T cell-astrocyte-complement-microglia axis in which inflammatory inputs lower the threshold for synaptic recognition and engulfment. If this response is excessive or insufficiently resolved, adaptive remodeling may shift toward pathological pruning and cognitive decline.

### 6.2 Microglia-synapse interactions in the perioperative brain

Microglial synaptic engulfment is normally restrained by inhibitory “don’t-eat-me” signals. The CD47-SIRPα pathway is particularly relevant because neuronal CD47 suppresses microglial phagocytic signaling through SIRPα (135, 136). Perioperative anesthesia and surgical stress can reduce hippocampal neuronal CD47 expression, weakening this protective threshold and increasing the likelihood that neurons or synaptic elements are aberrantly targeted for removal (137). This mechanism provides a direct link between perioperative stress, reduced synaptic protection, and pathological pruning. Anesthetic states may also reshape microglia-synapse contacts without requiring overt structural injury. During isoflurane anesthesia, reduced norepinephrine signaling can drive microglial processes deeper into synaptic regions, where they colocalize with GABAergic synapses and physically mask inhibitory inputs. This can trigger transient neuronal hyperactivity after anesthesia and depends on microglia and Adrb2 signaling (138–140). In resilient brains, such changes may be reversible. In primed or metabolically vulnerable brains, however, reduced inhibitory protection and abnormal microglia-synapse contacts may delay restoration of synaptic homeostasis, contributing to postoperative agitation, pain sensitization, or cognitive fluctuations.

### 6.3 Network-level consequences of perioperative neuroinflammation

Synaptic injury becomes clinically relevant when it disrupts circuit coordination. Perioperative neuroinflammation may impair inhibitory interneuron function and long-range synchronization, thereby destabilizing cognition-related rhythms (141). In aged or inflamed brains, parvalbumin-positive interneurons may be particularly vulnerable, and loss of microcircuit inhibition can reduce gamma power and rhythmic stability (142). When perineuronal net protection is insufficient, microglia-mediated synaptic stripping may further amplify excitation-inhibition imbalance (143).

Long-range hippocampo-cortical communication may also be affected. Surgery-induced neuroinflammatory transcriptional programs in the medial prefrontal cortex are associated with impaired excitatory synaptic transmission, inhibitory imbalance, reduced signal-to-noise ratio, and disorganized background firing (144). These changes may weaken hippocampal theta–prefrontal gamma coupling and reduce the prefrontal cortex’s selective readout of hippocampal inputs. Although these oscillatory mechanisms still require more direct validation in PND models, they provide a plausible bridge between microglial threshold lowering, delayed synaptic recovery, and postoperative deficits in attention, executive function, and recent memory.

## 7 Targeting neuroinflammation in a time- and state-dependent manner

**Figure 7:**
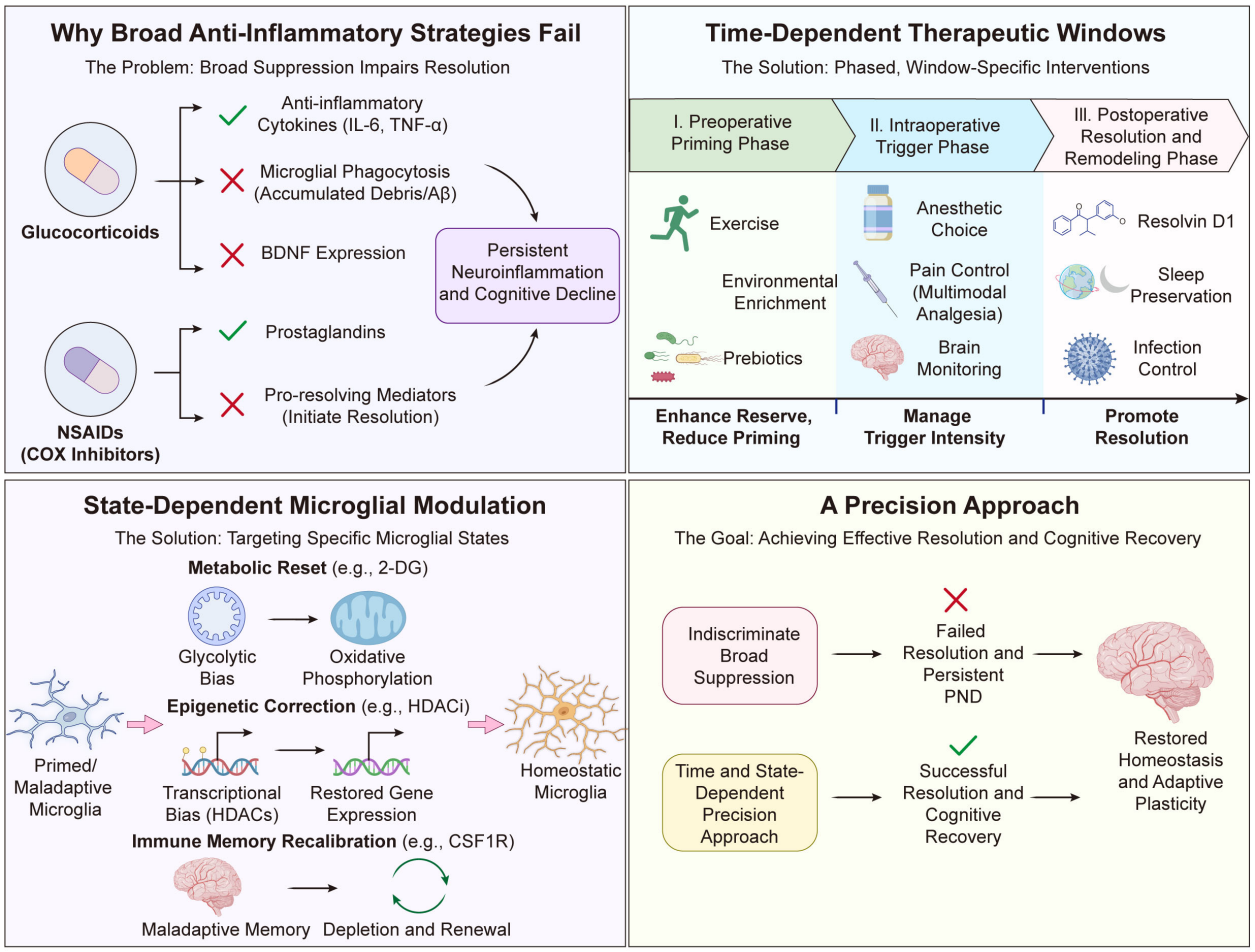
Precision anti-inflammatory strategies for perioperative neurocognitive protection. Although glucocorticoids or NSAIDs can reduce pro-inflammatory mediators, they may also suppress microglial clearance functions and pro-resolving signals, thereby delaying inflammatory resolution and contributing to persistent neuroinflammation and cognitive decline. A more rational approach is to implement phased, window-specific interventions by reducing priming and enhancing reserve preoperatively, limiting trigger intensity intraoperatively, and promoting resolution and remodeling postoperatively. This strategy can be complemented by state-dependent, precision modulation of microglia, such as metabolic resetting, epigenetic correction, or immune memory recalibration, to support cognitive recovery. Aβ, amyloid-β; BDNF, brain-derived neurotrophic factor; CSF1R, colony-stimulating factor 1 receptor; HDACi, histone deacetylase inhibitor; 2-DG, 2-deoxy-D-glucose.

Therapeutic strategies for PND have traditionally focused on suppressing postoperative neuroinflammation. However, reducing inflammatory mediators alone does not necessarily translate into cognitive protection. In cardiac surgery populations, randomized controlled trials (RCTs) have shown that dexamethasone can reduce serum C-reactive protein, but does not reduce delirium incidence within the first 4 postoperative days or shorten delirium duration (145). This suggests that the key pathological issue in PND is not only the magnitude of inflammation, but also whether the CNS can restore homeostasis after perioperative immune challenge. Broad anti-inflammatory strategies may interfere with microglial clearance, tissue repair, and pro-resolution programs, thereby delaying recovery rather than promoting it (**Figure 7**).

### 7.1 Why broad anti-inflammatory strategies fail in PND

Glucocorticoids and NSAIDs can suppress inflammatory mediators, but their clinical benefits for PND remain inconsistent (146–149). One explanation is that inflammation resolution is an active biological process rather than simple disappearance of cytokines. Glucocorticoids may reduce IL-6, TNF-α, and IL-1β, but they can also impair microglial phagocytosis, delay clearance of neurotoxic debris such as Aβ, and suppress hippocampal BDNF expression (150–152). Similarly, NSAIDs inhibit COX-dependent prostaglandin synthesis, but this may also limit substrates required for specialized pro-resolving mediator production (153, 154).

Therefore, broad immunosuppression may be insufficient or even counterproductive when delivered without regard to timing or microglial state. PND prevention should shift from indiscriminate inflammatory blockade toward strategies that preserve necessary defense and repair functions while promoting timely resolution.

### 7.2 Timing matters: therapeutic windows for perioperative intervention

PND develop across linked perioperative phases, including preoperative priming, intraoperative triggering, and postoperative resolution or maladaptive persistence. Each phase may require a different therapeutic priority.

#### 7.2.1 Window I: preoperative priming phase

Before surgery, the main goal is to reduce microglial priming and enhance cognitive and metabolic reserve. Representative approaches include preoperative exercise, environmental enrichment, and modulation of gut–brain signaling through prebiotic strategies (155–157). These interventions may improve mitochondrial fitness, increase BDNF-related resilience, and shift microglia toward a less pro-inflammatory baseline. Rather than treating established neuroinflammation, this window aims to raise the threshold for exaggerated perioperative responses.

#### 7.2.2 Window II: intraoperative trigger phase

During surgery, the priority is to reduce trigger intensity and prevent a primed brain immune landscape from shifting into excessive neuroinflammation. This includes careful anesthetic selection, stable physiological management, adequate analgesia, and avoidance of excessive sedative, anticholinergic, or opioid burden. Inhalational anesthetics such as sevoflurane and intravenous agents such as propofol may exert different neuroimmune effects, but their impact depends on dose, age, baseline vulnerability, and surgical context (158–163). Multimodal analgesia and individualized brain or physiological monitoring may further reduce nociceptive, hemodynamic, and network-level stress (164, 165).

#### 7.2.3 Window III: postoperative resolution and remodeling phase

After surgery, the therapeutic goal should shift from suppressing inflammation to promoting resolution, clearance, and neural network recovery. Representative strategies include pro-resolving mediators such as Resolvin D1, preservation of sleep architecture, optimization of analgesia and sedation, and prevention or early treatment of postoperative second hits such as infection, hypoxia, hyperglycemia, or metabolic disturbance (166–168). These measures may help guide microglia back toward homeostatic surveillance and prevent acute inflammation from becoming persistent or recurrent.

### 7.3 State-dependent modulation of microglial function

In addition to timing, intervention should be guided by microglial state. A primed, glycolysis-biased, epigenetically reprogrammed, or memory-like microglial state may require different forms of modulation. Representative strategies include metabolic resetting to reduce glycolytic bias, epigenetic correction to reverse maladaptive transcriptional programs, and immune-memory recalibration to restore response thresholds (106, 116, 169). These approaches remain largely preclinical or hypothesis-generating in the PND field. For example, glycolysis inhibitors such as 2-DG, class I HDAC inhibitors, and CSF1R-based microglial renewal strategies provide mechanistic proof-of-concept, but their perioperative use requires careful assessment of CNS delivery, timing, immune defense, infection risk, and patient selection. Future therapeutic development should therefore combine time-window selection with biomarkers that reflect baseline immune vulnerability, microglial state, and resolution capacity.

## 8 Conclusions and future directions

PND arise not from surgical inflammation alone, but from a mismatch between acute perioperative immune challenge and a pre−existing, microglia−centered brain immune landscape. This landscape is shaped by aging, preoperative cognitive impairment, comorbidities, and prior inflammatory exposure, and it determines the threshold for inflammatory initiation, the mode of amplification, and the capacity for resolution. Microglia serve as central integrators of peripheral−to−central immune signals, yet their perioperative responses are not binary but lie along a dynamic continuum of states. In a primed or vulnerable brain, microglia exhibit lower activation thresholds, exaggerated effector programs, and impaired resolution capacity—states sustained by durable reprogramming mechanisms including mitochondrial dysfunction, glycolytic bias, epigenetic remodeling, and trained immunity−like memory. These cell−intrinsic changes, together with multicellular interactions involving astrocytes, vascular cells, and infiltrating immune cells, determine whether postoperative neuroinflammation resolves or persists, leading to synaptic loss, network dysfunction, and heterogeneous cognitive outcomes. This framework explains why broad anti−inflammatory strategies often fail and argues instead for time−window−specific and state−dependent interventions: reducing preoperative priming and enhancing cognitive reserve, limiting intraoperative triggers, and actively promoting postoperative resolution and repair.

Future research should prioritize longitudinal, high−resolution mapping of microglial state trajectories across perioperative time points, combined with causal testing of metabolic and epigenetic reprogramming pathways such as mitochondrial quality control, glycolysis, histone modification, and trained immunity. Equally important is the dissection of multicellular networks *in vivo*, moving beyond a microglia−centric view to include astrocytes, endothelium, and peripheral immune cells. For clinical translation, biomarkers that reflect microglial homeostatic loss, metabolic reprogramming, or resolution capacity are urgently needed to stratify patients and enable time−locked, biomarker−guided trials. Finally, experimental models must systematically incorporate clinically relevant modifiers—aging, diabetes, obesity, hypertension, and preoperative cognitive impairment-as core determinants of the baseline immune landscape. By reframing PND as a disorder of microglial state transitions and resolution failure, this integrated perspective offers a roadmap for precision perioperative medicine aimed at preserving cognitive resilience and restoring neural network homeostasis.
